# Synchrotron Bragg diffraction imaging characterization of synthetic diamond crystals for optical and electronic power device applications^1^ ^1^

**DOI:** 10.1107/S1600576717003831

**Published:** 2017-03-29

**Authors:** Thu Nhi Tran Thi, J. Morse, D. Caliste, B. Fernandez, D. Eon, J. Härtwig, C. Barbay, C. Mer-Calfati, N. Tranchant, J. C. Arnault, T. A. Lafford, J. Baruchel

**Affiliations:** a European Synchrotron Radiation Facility (ESRF), Grenoble, France; b L Sim, MEM, UMR-E CEA/UGA, INAC, Grenoble, France; c Institut Néel, University Grenoble Alpes, Néel F-38000, France; d Institut Néel, CNRS, Grenoble F-38000, France; e University of Johannesburg, Auckland Park, South Africa; f CEA, LIST, Diamond Sensors Laboratory, Gif-sur-Yvette F-91191, France

**Keywords:** X-ray diffaction, topography, diamond, rocking curve imaging

## Abstract

Bragg diffraction imaging enables the quality of synthetic single-crystal diamond substrates and their overgrown, mostly doped, diamond layers to be characterized. This is very important for improving diamond-based devices produced for X-ray optics and power electronics applications. The usual first step for this characterization is white-beam X-ray diffraction topography, which is a simple and fast method to identify the extended defects (dislocations, growth sectors, boundaries, stacking faults, overall curvature *etc.*) within the crystal. This allows easy and quick comparison of the crystal quality of diamond plates available from various commercial suppliers. When needed, rocking curve imaging (RCI) is also employed, which is the quantitative counterpart of monochromatic Bragg diffraction imaging. RCI enables the local determination of both the effective misorientation, which results from lattice parameter variation and the local lattice tilt, and the local Bragg position. Maps derived from these parameters are used to measure the magnitude of the distortions associated with polishing damage and the depth of this damage within the volume of the crystal. For overgrown layers, these maps also reveal the distortion induced by the incorporation of impurities such as boron, or the lattice parameter variations associated with the presence of growth-incorporated nitrogen. These techniques are described, and their capabilities for studying the quality of diamond substrates and overgrown layers, and the surface damage caused by mechanical polishing, are illustrated by examples.

## Abstract 2

Bragg diffraction imaging techniques are described, and their capabilities for studying the quality of diamond substrates and overgrown layers, and the surface damage caused by mechanical polishing, are illustrated by examples.

## 1. Introduction   

High-quality single-crystal diamond has many attractive physical properties, in particular its wide band gap and very high thermal conductivity, charge mobility and breakdown electric field strength (Chicot *et al.*, 2014 ▸; European Horizon 2020 GreenDiamond project, http://www.greendiamond-project.eu). We are concerned with the use of diamond in the fields of electronics, detectors and X-ray optics. Promising results have been obtained for high-voltage diodes fabricated with boron-doped diamond (Chicot *et al.*, 2014 ▸) and various electronic devices are now being developed to commute high power at high frequency in order to minimize losses in power distribution networks (Horizon 2020 GreenDiamond project, http://www.greendiamond-project.eu). In another application, high-quality (111) surface orientation diamond plates are needed at synchrotron light sources to be used as X-ray beam splitters, phase plates and monochromators (Shvyd’ko *et al.*, 2011 ▸; Van Vaerenbergh *et al.*, 2010 ▸). Extremely brilliant X-ray beams are produced at synchrotrons using undulator insertion devices, which are massive structures, several metres in length, equipped with spatially alternating magnetic fields. It has been theoretically shown (Korol *et al.*, 2014 ▸) that a far more compact undulator can be produced using a structured diamond crystal which has layers of boron doping to create tailored strain patterns in its crystal lattice. Undulator crystals based on this innovative fabrication method could lead to the generation of intense monochromatic radiation in the γ region of the energy spectrum (Backe *et al.*, 2013 ▸).

All the above applications require diamond plates with the main surface orientation (100) and/or (111), with a low miscut angle (less than ∼0.1°), and with highly perfect bulk and surface crystal quality. In addition, these diamonds should be available with large areas (several 10 mm^2^) free of extended defects such as dislocations. Commercially available single-crystal diamond substrates currently exhibit a wide variety of crystal qualities, depending on the producer and the price.

X-ray Bragg diffraction imaging (X-ray topography) is a long-established technique for characterizing crystal defects (Authier, 2001 ▸). Many of these techniques have been implemented on the BM05 beamline at the ESRF: they produce images of crystal defects *via* the associated distortion field induced in the crystal lattice, revealing their locations over large sample areas (>100 mm^2^), while at the same time providing spatial resolutions down to the micrometre scale (Klapper, 1980 ▸; Bowen & Tanner, 1998 ▸; Authier, 2001 ▸).

Historically, diamond is one of the materials most extensively investigated by Bragg diffraction imaging. Lang published a series of topographs of natural diamonds as early as 1963 (Lang, 1963 ▸) and an excellent review of the work performed using X-ray diffraction topography on diamonds in 1974 (Lang, 1974 ▸). The work was pursued by Lang and many other teams around the world, on both natural and synthetic diamonds. A recent review of the defects in synthetic diamonds has been published by Moore *et al.* (2016 ▸).

The original contribution of the present work is its use of complementary synchrotron-based Bragg diffraction imaging techniques, going from simple white-beam topography to sophisticated rocking curve imaging, in order to characterize a large series of commercially supplied diamond plates. White-beam images reveal, in a fast and efficient manner, the samples’ bulk crystalline quality, together with some surface features like polishing-induced damage. As required, a higher sensitivity to defects and quantitative results can then be inferred from additional rocking curve imaging data. The present work demonstrates the capabilities of the Bragg diffraction imaging techniques we use, and gives insights into the state of the art of commercial diamond single-crystal substrates and overgrown layers.

## 2. Description of the samples   

A series of (100) surface-oriented diamond crystals was characterized in the present study. They originated from various commercial sources (see Table 1 ▸) and displayed markedly different crystal qualities. They either are simple substrates (0.5 mm thick) or include overgrown layers. We note that when fabricating devices attention must be paid to both the quality of the doped overlayers, which are grown by microwave-assisted plasma chemical vapour deposition (MWCVD), and the quality of the high-temperature high-pressure (HPHT) substrate that is used as the growth template: defects present in the bulk, such as dislocations, stacking faults, growth sector boundaries and defects introduced by surface processing (laser cutting and abrasive polishing), may all play an important, often detrimental, role in the performance of the final fabricated device.

Samples 1–6 are single-crystal diamond plates delivered without any post-abrasive polishing surface processing other than hot acid cleaning. Samples 1 and 2 were grown by the CVD method, supplied by Element Six Ltd (e6), England, and Excellent Diamond Products Co. (EDP), Japan, respectively. Samples 3 and 4 are HPHT-grown type Ib samples, a type usually used as a substrate for CVD overgrowth, supplied by Sumitomo Electric Carbide Inc. (Sumi), Japan, and IIa Technologies Pte. Ltd (IIa-Tech), Singapore, respectively. Samples 5 and 6 are high-crystalline-quality HPHT type IIa samples, supplied by New Diamond Technology LLC (NDT), Russia, and e6, respectively.

A second series of samples employed for this study consisted of diamond plates overgrown by the CVD method with one or more boron-doped diamond layers. Sample 7 is a 0.7 mm thick HPHT substrate which was overgrown with a 0.7 µm thick CVD boron-doped layer ([B] ≃ 10^21^ atom cm^−3^). The doping concentration of the boron-doped layers was measured by secondary ion mass spectrometry (SIMS). We needed to characterize the crystalline quality of Sample 8, prepared for use as an undulator crystal. The fabrication of a diamond undulator involves varying the concentration of boron in the plasma during MWCVD growth (the detailed properties of such a device will be discussed in a separate future publication). The boron doping leads to an expansion of the diamond lattice, and so a periodic variation in the lattice parameter can be introduced with a suitable variation in concentration of the substitutional boron impurity atoms during layer growth. Our expectation was that high crystal lattice quality, *i.e.* bulk crystallinity, would be retained up to a boron/carbon atom concentration ratio of ∼1.5% (Backe *et al.*, 2013 ▸; Korol *et al.*, 2014 ▸). A test diamond superlattice was produced at the Institut Néel. It consisted of a four-period graded-concentration B-doped diamond layer grown on a high-quality type IIa substrate, with a boron concentration varying from 2 × 10^20^ to ∼8 × 10^20^ atom cm^−3^ (corresponding to ∼3000 p.p.m.). The thickness of each period was 3.5 µm.

Sample 9 is a (100) surface HPHT type Ib diamond that was overgrown with a 25 µm thick diamond layer, nominally not doped with boron (<10^15^ atom cm^−3^), using the MWCVD process at the CEA laboratory.

## 3. X-ray diffraction topography techniques: white-beam and rocking curve imaging   

**Figure 1:**
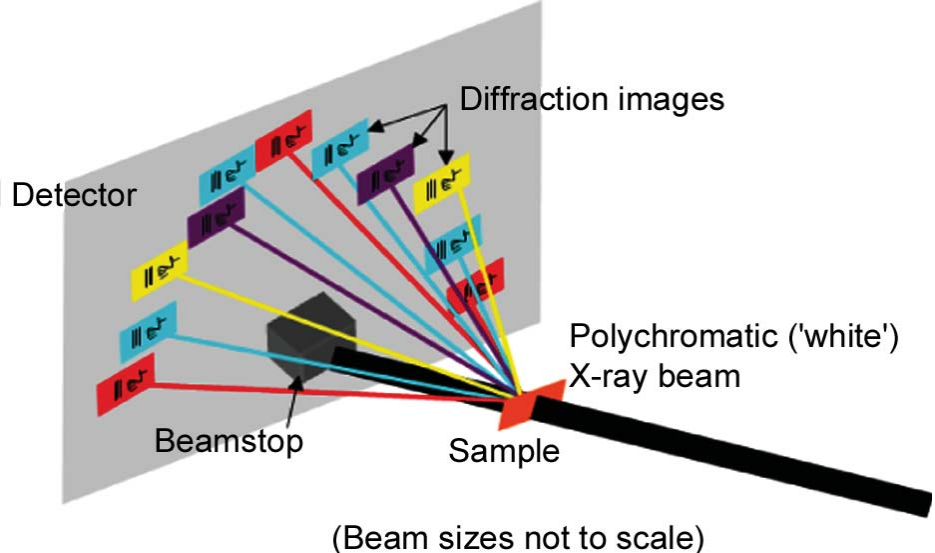
Scheme of white-beam topography.

The simplest synchrotron-based X-ray Bragg diffraction imaging approach is white-beam X-ray topography. The sample is placed in a polychromatic beam which is, if possible, larger than the sample. This produces a Laue pattern, where multiple Bragg diffraction spots are generated (Fig. 1 ▸). The synchrotron beam has a low divergence, so each spot is a Bragg diffraction image (topograph) of the illuminated sample volume and shows intensity variations associated with defects that have distorted the sample’s crystal lattice (Klapper, 1980 ▸; Bowen & Tanner, 1998 ▸; Authier, 2001 ▸). These topographs are usually recorded on high-spatial-resolution (∼1 µm) X-ray sensitive film. Both the general shape of the topograph and the contrast (*i.e.* intensity variations) observed within each diffraction spot provide information on the distortions of the crystal lattice planes producing the particular topograph. The image expected from a perfect rectangular-shaped crystal, illuminated over its entire surface by the X-ray beam in transmission mode and with the detector perpendicular to the incoming beam, is itself simply a rectangle, with the projected dimensions of the area illuminated by the beam, as shown schematically in Fig. 1 ▸.

If the crystal is not perfect, the lattice distortions lead to intensity variations within the topograph. For the low X-ray absorption case with which we are concerned, an additional diffracted intensity results from lattice distortion gradients associated with such defects, for example dislocations. Macroscopic curvature of the sample or local distortions of the crystal (subgrain boundaries, cracks *etc.*) are often associated with significant modification of the overall outline shape of the topographic image.

**Figure 2:**
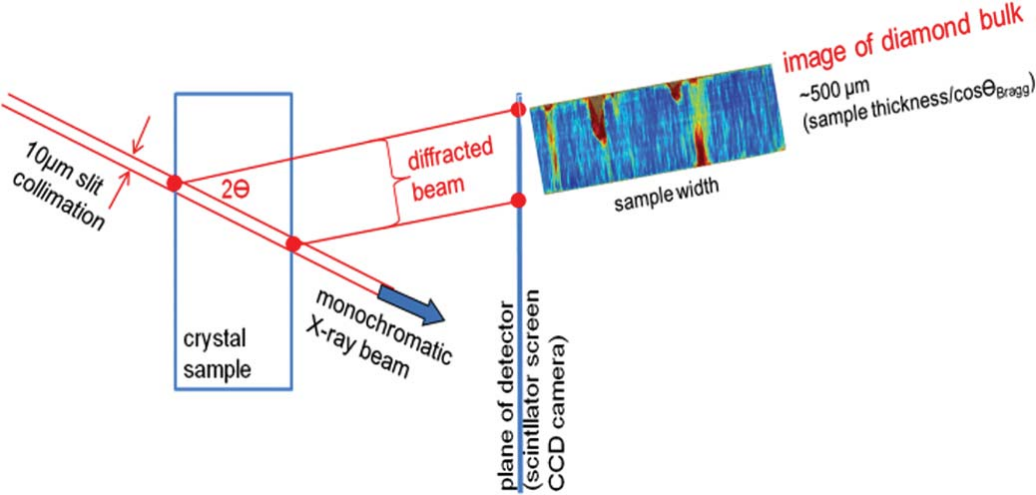
Geometry of section topography. A ∼10 µm wide strip beam is used to characterize a cross section of the diamond plate through its thickness. For clarity, the multi-strip beam geometry is not shown.

In the usual projection version of Bragg diffraction imaging (Lang, 1959 ▸), where the entire sample is illuminated by the beam, the images projected onto the two-dimensional film detector are the integrals of the diffraction contributions arising from the entire thickness of the sample, *i.e.* information on the depth location of the defect(s) is lost because a three-dimensional volume is projected onto a two-dimensional surface. When it is important, as in our case, to distinguish between defects at the near surface and those in the bulk of the crystal, section topography (Lang, 1958 ▸) must be used. Section topography may be carried out in both white and monochromatic beams. In this technique, the incident beam is restricted in one dimension by a narrow collimation slit to a strip about 10 µm wide. The intersection of the 10 µm wide strip beam with the crystal can be considered as creating a virtual two-dimensional slice. As can be understood by reference to Fig. 2 ▸, one side of this section image projected onto the two-dimensional detector corresponds to the side where the X-ray beam enters the crystal sample, and the other side corresponds to where the beam exits the sample. This technique drastically reduces the sample volume investigated by the beam, but in practice this drawback is partly overcome by using a multi-strip beam which enables images of multiple sections of the sample to be recorded simultaneously at different locations across the sample surface. A 25 µm thick gold mask including 10 µm slits separated by 0.5 mm was used to produce this multi-strip beam.

**Figure 3:**
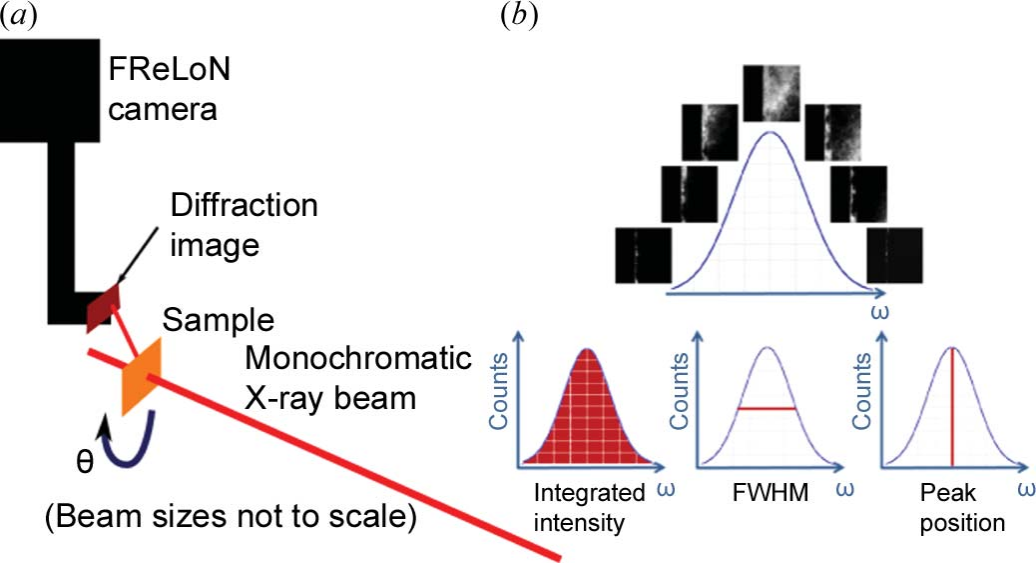
(*a*) A schematic diagram of Bragg diffraction imaging using the RCI technique with a monochromatic incident beam. (*b*) Acquisition of an image series with calculation of integrated intensity, FWHM and diffraction peak position, extracted on a pixel-by-pixel basis from the local rocking curves.

The rocking curve imaging (RCI) technique is a quantitative version of double-crystal topography. By using digital cameras together with sophisticated analysis software, RCI enables the measurement of the spatial distribution of local lattice distortions. The RCI technique can be used in both reflection (Bragg) and transmission (Laue) geometries. Developments have been reported using the Laue geometry carried out in both projection (Lübbert *et al.*, 2000 ▸, 2005 ▸; Hoszowska *et al.*, 2001 ▸; Calamiotou *et al.*, 2007 ▸) and section (Kluender *et al.*, 2011 ▸; Pernot *et al.*, 2010 ▸; Tran Thi *et al.*, 2015 ▸) modes. Information is extracted on a pixel-by-pixel basis from a series of typically several hundred X-ray diffraction images recorded at small angular steps of the crystal rotation or rocking angle [θ in Fig. 3 ▸(*a*)] from one side to the other of a Bragg diffraction peak (Fig. 3 ▸ *b*). Maps of the integrated intensity, the FWHM and the peak position angle of the diffracted intensity are then calculated from the ensemble of these rocking curve images, using rocking curve imaging analysis (RCIA) software (T. N. Tran Thi, ESRF) which produces two-dimensional maps on a pixel-by-pixel basis. These maps provide quantitative values related to the local distortion of the crystal lattice.

## 4. Experimental results   

### 4.1. White-beam X-ray Bragg diffraction imaging   

White-beam diffraction topography images were recorded on the ESRF bending magnet beamline BM05 using X-ray films (Agfa Structurix D3-SC, 130 × 180 mm) with a sample-to-film distance of 300 mm. The beam size was typically collimated to 5 × 6 mm in order to use the most homogeneous part of the incident beam, *i.e.* to ensure a quasi-uniform intensity illumination of the whole diamond surface. The beam is not truly ‘white’ but has a continuous energy spectrum covering ∼10–120 keV. All the experiments were done in transmission (Laue) mode.

**Figure 4:**
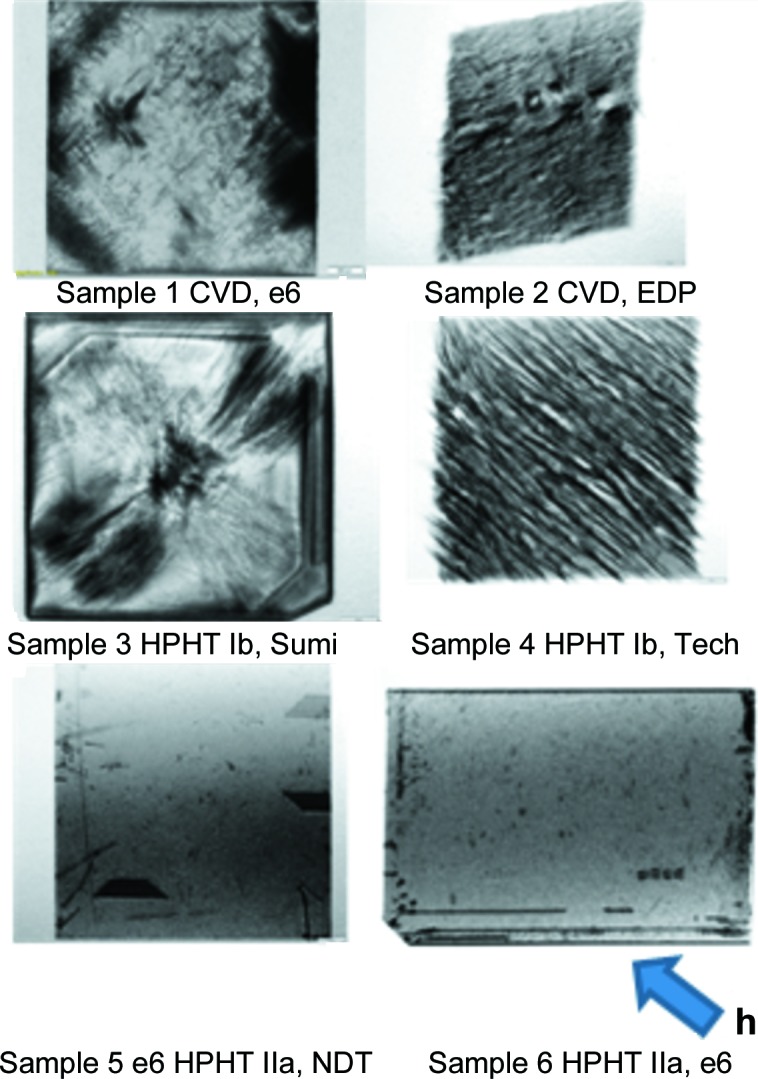
White-beam topographs of single-crystal diamond plates characterized at BM05. All the diamond plates are 3 × 3 mm, except Sample 6 which is 4 × 6 mm. The projection of the 220 diffraction vector, **h**, on the plane of the image is indicated by a blue arrow.

Fig. 4 ▸ shows white-beam topographs of the single-crystal diamond Samples 1 to 6 from Table 1 ▸. These topographs show clearly, through the differing contrast in the images, a wide sample-to-sample variation corresponding to the variation in crystal quality. The regions of higher diffracted intensities are the darker areas in the images (this presentation is the established convention), which in the low X-ray absorption case we are concerned with here are associated with distorted regions and lattice defects. All the images in Fig. 4 ▸ have a diffraction vector pointing in the [220] direction; the projection of this diffraction vector on the plane of the image is indicated by the blue arrow. The images of Samples 1 and 3 in Fig. 4 ▸ reveal dislocations lying along the 〈111〉 directions, as usually found for diamond, and as could be concluded from a comparison of the images recorded simultaneously for the 220, 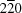 and 400 reflections (not shown here). For Sample 3, the dislocations are observed as individual black lines radiating mainly from the centre of the sample, this region being that above the seed crystal used for the HPHT growth.

The slowly grown type IIa crystals of Samples 5 and 6 clearly have the best quality: they both display large areas, within the [001] central growth sector, which are free from extended defects like dislocations in the bulk, and which exhibit only weak contrast features related to surface imperfections. Stacking faults are observed, as trapezoidal-shaped areas, at the periphery of Sample 5.

The low-grade CVD- and HPHT-grown plates display dislocations with densities estimated in the range 10^3^–10^4^ cm^−2^. The square Sample 2 produces a topograph that does not retain the outline shape of this crystal: the lozenge-shaped image indicates an overall distortion of the entire sample, as a result of stress associated with processing (*i.e.* cutting and polishing) into a flat plate, or more likely acquired during sample CVD growth. This extreme curvature was not apparent in any of the other diamond samples we measured.

A series of diagonal stripes with an orientation approximately along the diagonal [110] direction is visible in Sample 4. These could also be related to the supplier company’s particular laser cutting process. Unfortunately, the large contrast generated by these defects hides any observation of other bulk defects.

**Figure 5:**
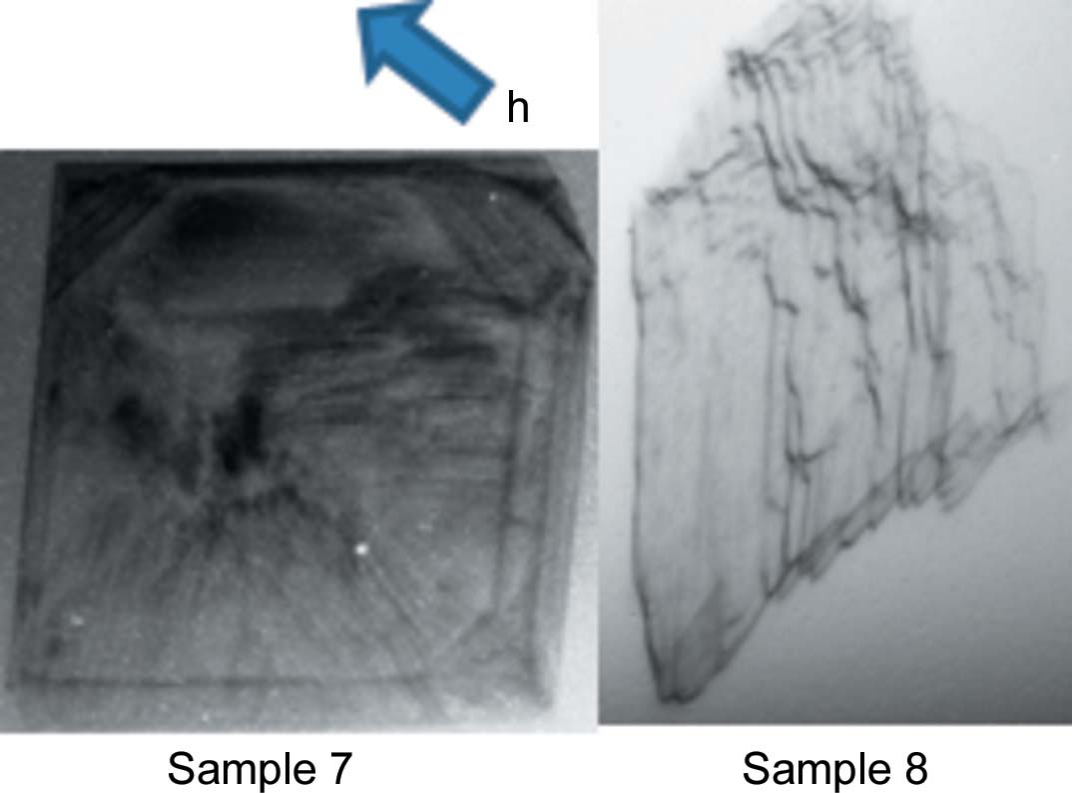
White-beam topographs of diamond substrates (3 × 3 mm) with MWCVD overgrown boron-doped diamond layers. The samples were completely illuminated by a square beam; the distorted shapes of the topographs result from overall curvature of the samples. The projection of the 220 diffraction vector, **h**, on the plane of the image is indicated by a blue arrow.

White-beam topography was also carried out on Samples 7 and 8 (Fig. 5 ▸), each of which had been overgrown with a boron-doped CVD layer. It is difficult to grow layers of high-quality crystal with boron doping levels >2 × 10^21^ atom cm^−3^ (Pernot *et al.*, 2010 ▸). Sample 7 has a doping level which is not far from this solution limit, but when comparing the details in the topograph of Sample 7 before and after growth, we observed no overall curvature and few additional defects produced during the growth process. This encouraging fact, which is in keeping with the electrical properties of diamond grown with high dopant concentrations reported in the literature (Tsoutsouva *et al.*, 2015 ▸), could be associated with the recent improvement of the MWCVD growth reactor at the Institut Néel where the doped layer was overgrown. The dislocations shown in the topograph are typical of the quality of the BULK Sumi Ib substrates used.

While there is only a slight image shape change for Sample 7, the shape of the topograph of Sample 8 (completely illuminated by the incoming beam) is dramatically distorted. As indicated above, with a thin (∼300 µm) sample and a detector nearly parallel to the main sample surface, shape distortion does not result from the projection geometry, but corresponds to the curvature of the sample itself. The thickness and concentration of the overgrown boron-doped layers (as measured by SIMS, see Table 1 ▸) clearly influence the global curvature of the samples. The deformation of the entire diffraction image to a parallelogram-like shape corresponds to the overall curvature of the sample. In addition to this global distortion caused by the 14 µm thick boron-doped overgrown layer, we observe a strong inhomogeneity in this sample, shown by the missing top-right part of its diffraction image. The HPHT bulk substrate of this sample was of high crystalline quality, verified by X-ray topography to be almost dis­location-free prior to the boron-doped layer overgrowth. This suggests that the CVD growth process was non-homogeneous across the surface, and the chaotic features suggest that either a chemical inhomogeneity (like a spatial variation in the amount of boron incorporated) or a structural inhomogeneity (which could include the layer thickness variation) occurred during the deposition process.

### 4.2. Monochromatic rocking curve imaging   

To obtain more precise quantitative information about the deformations in diamond, we used a beam produced by a double-crystal Si(111) monochromator at an energy of 20 keV. Bragg diffraction images were acquired on a detector system comprising a scintillator screen equipped with microscope folded-relay optics to the ESRF-developed 2048 × 2048 pixel FReLoN CCD camera (Labiche *et al.*, 2007 ▸). The optical demagnification of the CCD sensor pixels in this system resulted in an effective square pixel size of 0.7 µm at the scintillator screen, so the recorded images had a corresponding field of view of ∼1.4 × 1.4 mm^2^. X-ray topographs of the 004 reflection were recorded using the RCI technique described above, *i.e.* multiple images were acquired while rotating the sample with respect to the beam axis, in this case with angular steps of ∼10^−4^° through the Bragg diffraction angle (Figs. 2 ▸ *a* and 2 ▸ *b*). The diffraction plane was vertical, and the distance between the sample and the detector scintillator screen was 50 mm.

The resultant FWHM and Bragg peak angle position (PPOS) RCI maps reveal different aspects of the local effective misorientation, Δθ, which is composed of two main contributions, the tilt of the lattice planes δθ and the lattice parameter variation Δ*d*/*d*, as
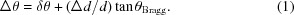
The RCIA program was employed to produce the image data sets. Each local rocking curve (corresponding to a single pixel in the series of CCD images) is fitted independently using a Gaussian function, *i.e.*

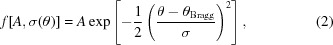
where *A* corresponds to the peak diffracted intensity observed at the Bragg angle, θ_Bragg_ is the angle of maximum intensity (*i.e.* the local Bragg angle in this pixel) and θ is the varying sample angle. The local FWHM (= 2.35σ) is representative of the local lattice quality of the crystal sample.

**Figure 6:**
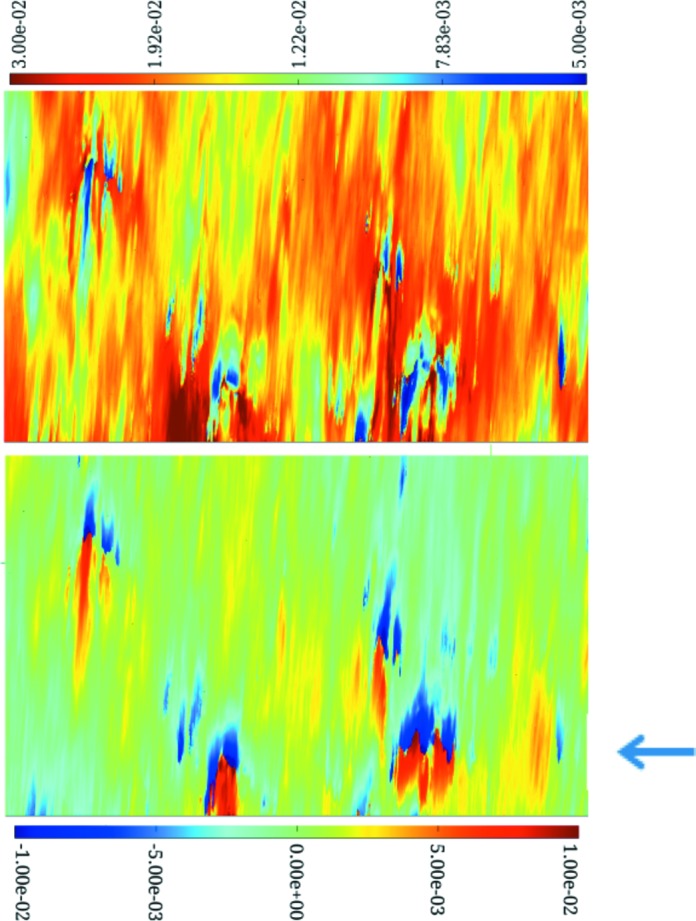
(Top) FWHM and (bottom) peak position maps (in degrees) of a low-quality CVD single crystal (Sample 2) with surface polishing damage. The images correspond to an area of the diamond sample of 0.7 × 1.4 mm. The blue arrow corresponds to the location of the central section topograph of Fig. 8 ▸.

Usually, comparison of the integrated intensity (INT), the FWHM and the PPOS maps around a given defect shows, in the low-absorption X-ray case, that the defect is associated both with an increase in the local integrated intensity and local FWHM, and with a variation in the peak position. This is the case, for instance, for dislocations, where variations in the peak position of ∼4 × 10^−4^° were measured on either side of the dislocation seen in the image in Fig. 6 ▸ (Tsoutsouva *et al.*, 2015 ▸). However, we must be cautious when interpreting RCI projection maps in transmission mode with thick crystals, because they result from diffracted intensity integrated over the X-ray path throughout the crystal thickness.

**Figure 7:**
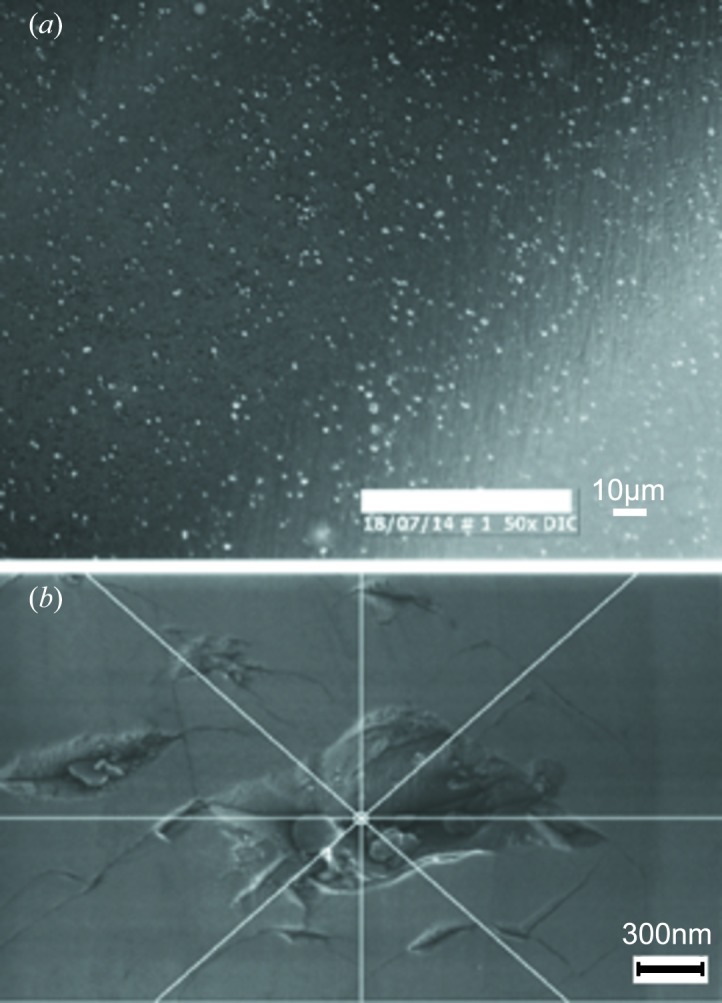
A diamond surface polished using a resin wheel. (*a*) Differential interference contrast optical microscopy, showing a distribution of micrometre-scale surface pits (seen here as bright spots). (*b*) Scanning electron microscopy (SEM) detail of a single pit, showing the cracks resulting from the abrasive polishing damage. (SEM image courtesy of E. Bustarret, Institut Néel, Grenoble.)

To illustrate the capabilities of the RCI technique, we choose here to show images corresponding to one of the low-quality diamonds, where interpretation requires the combination of both projection and section diffraction images. For the projection topography FWHM map (Fig. 6 ▸) of Sample 2, the less distorted (blue) areas correspond to an FWHM of ∼5 × 10^−3^–10^−2^°. This is more than an order of magnitude higher than the FWHM expected for a nearly perfect diamond crystal. The corresponding PPOS map of the same region shows that rapid peak-position variations do not occur, as might have been expected, for the higher FWHM values, but do occur for the lower FWHM values. This apparent contradiction is resolved by section RCI, which gives access to quantitative depth-resolved data, and by taking into account the polishing process employed for this sample. Sample 2 was polished by a resin wheel abrasive method which gives a surface roughness with an *R*_a_ figure (as measured by atomic force microscopy) in the range of a few nanometres. However, as shown in Figs. 7 ▸(*a*) and 7 ▸(*b*), this polishing method leaves a surface with pits which result from the impact damage of grit breakout from the resin wheel, and these pits are accompanied by deep crack damage extending into the bulk (Fig. 7 ▸ *b*).

**Figure 8:**
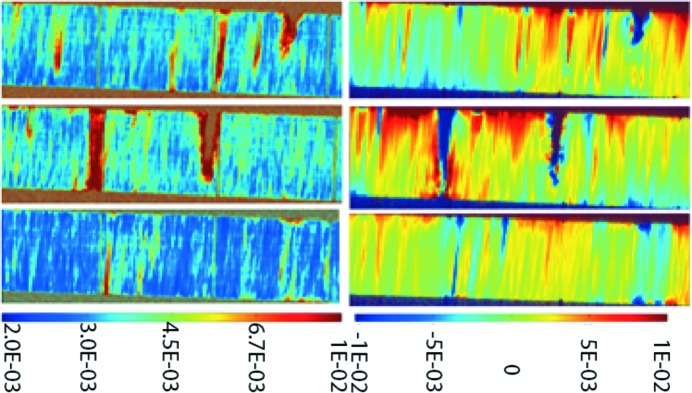
Multiple 400 reflection maps (in degrees) of (left) FWHM and (right) PPOS at different places across a diamond surface, obtained using section topography RCI. The colour scale shows the variation in crystalline quality in a virtual cross section through the ∼500 µm thick diamond plate of Sample 2. The size per image is ∼1.4 × 0.5 mm (vertical beam height × horizontal sample thickness).

Using the method described in Fig. 2 ▸, section RCI maps were recorded across the surface of Sample 2 using multiple strip beams of width 10 µm spaced 0.4 mm apart. Fig. 8 ▸ shows the series of calculated section RCI FWHM and PPOS maps that were obtained. The location of the central section is indicated in Fig. 6 ▸ by an arrow. The images show, in the vertical direction, the variation in FWHM and peak position through the ∼0.5 mm thickness of the sample. We note several features:

(i) The blue areas in the section image, which mainly correspond to the diamond bulk, display an FWHM of ∼3 × 10^−3^°, which is far less than that observed in the projection FWHM RCI (Fig. 6 ▸, top).

(ii) A region of higher FWHM (∼4 × 10^−3^°), about 20 µm thick, is observed on the upper border of the image, corresponding to one of the polished surfaces.

(iii) An overall variation in the Bragg peak position of ∼3 × 10^−3^ to 10^−2^° is observed between the two surfaces; this corresponds to a radius of curvature of the lattice planes concerned of less than 10 m.

In addition, in both the FWHM and PPOS maps, empty non-diffracting areas (brown in the FWHM figure) are observed: these correspond to deep ‘holes’ starting from one of the surfaces. The larger of these holes are >30 µm in diameter at the surface and >250 µm in depth. They are surrounded by areas of large FWHM (∼10^−2^°) and in the PPOS maps by areas where the peak position changes rapidly (∼10^−2^°). These holes are not physical voids throughout the bulk, but we interpret these apparent holes as seen in the diffraction data as resulting from the real damage pits observed in the surface.

Let us now come back to the occurrence, pointed out above, of areas with small FWHM and quickly varying peak position shown in Fig. 6 ▸. Assuming, as a first approximation, a kinematical behaviour, which is justified when taking into account the level of local FWHM (about one order of magnitude above the intrinsic diffraction width of perfect diamond) and considering the overall curvature of the lattice planes when going from one surface to the other, the measured projection RCI FWHM results from the convolution of two terms: the local FWHM (∼3 × 10^−3^°, as indicated by the FWHM section RCI) and the variation in peak position along the path of the X-rays reaching a given pixel of the detector (∼10^−2^°, as indicated by the PPOS section RCI). This latter term is, in our case, the dominant one. This diffracting region/path in the sample is reduced when a hole is present along this path. The remaining region that diffracts into the pixel we are concerned with exhibits a reduced X-ray path, and consequently a reduced variation in the peak position and FWHM. This simple geometric approach explains for the most part the measured values in Fig. 6 ▸.

The distortion caused by the observed holes is not equal on both sides of the diamonds. Fig. 8 ▸ shows three section topographs obtained using the gold multi-slits described above. These images correspond to three 10 µm thick virtual slices, located 0.5 mm away from each other. They show that the holes and their associated distortion are greater on the upper surface. In some surface areas there are no holes present, but we observe, nearly everywhere in the bulk, red or yellow filaments running from one side to the other of the FWHM section images. These are, as discussed above, associated with surface damage distortion that propagates throughout the whole crystal thickness, and also with dislocations in the bulk generated during sample growth which are aligned predominantly with the CVD (100) growth direction (Tsoutsouva *et al.*, 2015 ▸).

**Figure 9:**
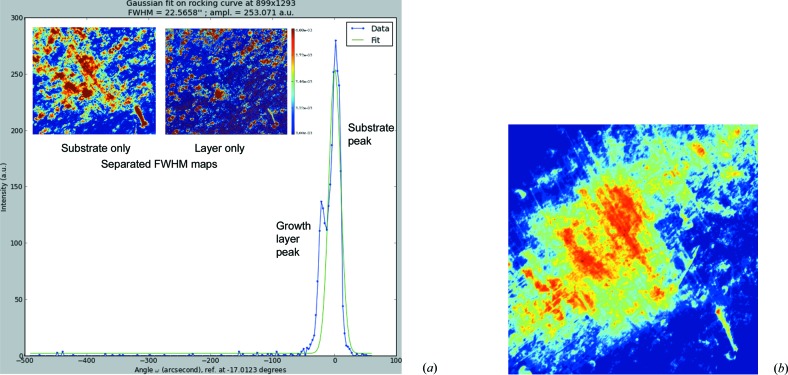
(*a*) Rocking curve of the CVD overgrowth layer on an HPHT substrate and extracted FWHM maps (in degrees) fitted separately for substrate and layer. (*b*) An individual image from the set generated by the RCI technique.

Another example where the RCI technique is very useful for the characterization of diamond is the study of the overgrown boron-doped diamond layers required for the fabrication of electronic devices. The quality of these layers is of great importance to the electrical performance of high-power MOS transistors currently being developed (Chicot *et al.*, 2014 ▸). Fig. 9 ▸(*a*) shows data computed from 360 rocking curve images of a (001) HPHT type Ib diamond that was overgrown with a 25 µm thick boron-doped diamond layer using the MWCVD process. The exact growth conditions are confidential. The 400 reflection rocking curve of Fig. 9 ▸ shows two peaks, separated by 7 × 10^−4^°. The stronger peak is from the diamond substrate and the weaker peak from the overgrown layer. By Gaussian fitting and deconvolution of these two peaks, we were able to compute the separate peak FWHM maps corresponding to the substrate only and the overgrown layer only. The insets of Fig. 9 ▸(*a*) show these maps: the FWHM of the substrate appears, on these maps, to be on average higher than that of the substrate, but we cannot extract simple conclusions from this experimental result, which is probably just related to the fact that the substrate is much thicker than the layer, and that the images correspond to an integration over the depth of the substrate or layer. On the other hand, we know that the substrate was cut perpendicularly to the growth direction from a region of the as-grown diamond located not very far from the seed. It is therefore expected that defects propagating from the seed would cross the platelet-shaped substrate from one of the main surfaces to the other and propagate to the layer. This is not observed. In particular, defects along the [110] direction are not present in the overgrown layer which was grown in the orthogonal [001] direction. Conversely, some of the defects observed in the overgrown layer do not correspond to those in the substrate. A simple way of estimating the relative number of these defects generated during overgrowth is just to subtract the two images of Fig. 9 ▸(*a*). The resulting difference image shows that the ‘uncommon’ defects cover only ∼10% of the total surface, suggesting a high quality of the growth method.

In addition to exploiting maps of diffraction peak intensity, peak position and peak FWHM, it is useful to investigate individual images from the set generated by the RCI technique. Fig. 9 ▸(*b*) shows such a diffraction image taken from the RCI set of 360 images, here halfway down from the peak of the rocking curve. We see, particularly in the less illuminated regions, straight features lying close to the [110] and [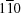] directions, which strongly suggest a network of misfit dis­locations with an average separation of ∼16 µm.

Let us try to correlate the various results presented in Fig. 9 ▸. As the experiment was performed in the symmetric transmission setting, a sensible assumption could be that no tilt is expected between the (001) planes of the substrate and the layer. Within this assumption, the angular variation between the two peaks originates only from the variation in lattice parameters and corresponds to Δ*d*/*d* ≃ 3 × 10^−5^. This variation is too high to be associated with a variation in dislocation density and therefore probably corresponds to a variation in dopant levels. Boron contamination of the intentionally boron-free overgrown layer was discarded: as Vegard’s law applies for low concentrations (<3 × 10^20^ atom cm^−3^), the measured Δ*d*/*d* would correspond to a boron concentration >10^19^ atom cm^−3^ (Brunet *et al.*, 1998 ▸). This value is orders of magnitude higher than any boron concentration expected to result from the growth process, where particular attention was paid to prevent such contamination. The Δ*d*/*d* value appears to be, in our case, mainly related to the variation in lattice parameter between the Ib HPHT substrate, known to contain between 100 and 200 p.p.m. of nitro­gen, and the overgrown layer, where no nitro­gen was added during growth. Indeed the lattice parameter variation we measured, again within the assumption of no or very small tilt of the (001) planes between the substrate and the layer, corresponds to 120 p.p.m. of nitro­gen (Voronov & Rakhmanina, 1997 ▸), assuming that the effect of any other impurities is negligible.

Misfit dislocations relax the elastic energy associated with this Δ*d*/*d* when the layer thickness is larger than the critical thickness. The layer thickness is 25 µm, which is about three times the critical thickness estimated using the Poisson ratio given by Klein & Cardinale (1993 ▸). The measured Δ*d*/*d* would lead, for a fully relaxed layer, to a mean distance between misfit dislocations of ∼12 µm, which is in reasonable agreement with the experimentally observed value.

## 5. Conclusions   

X-ray diffraction imaging (topography), in the version developed by Andrew Lang, has been very useful to help characterize the defects present in silicon crystals produced for the microelectronics industry. Today, new industrial challenges for X-ray diffraction topography arise, including, for instance, the characterization of synthesized diamond crystals and of defects in silicon crystals grown by low-cost methods and intended for photovoltaic purposes. To respond to these needs, we have developed synchrotron diffraction imaging techniques over the past few years that can provide quick qualitative evaluation of the crystal quality or (with RCI, both projection and section) provide quantitative maps of the local lattice distortions (local FWHM and peak angular position). The combination of projection and section RCI methods enables quantification of the local distortion as a function of depth in the crystal, through the investigation of virtual slices of the sample. Distortions can be measured with a precision in the microradian range. This work has shown that the use of these techniques allows full characterization of sample quality.

The diamonds investigated in the present work displayed a wide variety of crystal quality, largely corresponding to that declared by the suppliers. Demanding applications (in particular for X-ray diffractive optics) require detailed characterization as described in the present work, because only a fraction of the available commercial samples may show adequate quality for the intended application. We also note here that some of the low-cost commercial polishing processes are detrimental for many applications.

To conclude, let us note that the examples shown here, such as the imaging of induced damage (holes, cracks) resulting from a polishing process, the visualization and distinction between defects in an HPHT substrate and those arising in an overgrown CVD layer, and the quantitative analysis of the change in lattice parameters associated with boron dopant concentration, clearly indicate the unique capabilities of RCI. The combination of these diffraction imaging techniques constitutes an invaluable non-destructive tool for characterizing diamond, both for the bulk crystals required for radiation detectors and X-ray optics devices, and for the subsequently overgrown doped layers that are required for the fabrication of new power electronic devices.

## Figures and Tables

**Table 1:** The types of diamond sample measured and their corresponding commercial suppliers

Sample	Substrate type and supplier	B-doped CVD overgrowth
1	CVD, e6	No
2	CVD, EDP	No
3	HPHT Ib, Sumi	No
4	HPHT Ib, IIa-Tech	No
5	HPHT IIa, NDT	No
6	HPHT IIa, e6	No
7	HPHT Ib, Sumi	0.7 µm, [B] ≃ 10^21^ cm^−3^
8	HPHT IIa, e6	14 µm, [B] ≃ 2–8 × 10^20^ cm^−3^
9	HPHT Ib, Sumi	25 µm, [B] < 10^15^ cm^−3^
